# Effectiveness of the Training Given According to Self-Care Deficit Nursing Theory in the Prevention of Peristomal Skin Complications

**DOI:** 10.3390/nursrep16050155

**Published:** 2026-05-05

**Authors:** Ali Ay, Hülya Bulut

**Affiliations:** 1 Department of Surgical Nursing, Faculty of Health Science, Bingol University, 12000 Bingöl, Türkiye; 2 Department of Surgical Nursing, Faculty of Nursing, Gazi University, 06560 Ankara, Türkiye; bhulya@gazi.edu.tr

**Keywords:** peristomal skin complications, self-care deficit nursing theory, patient education, nursing care, tele-nursing

## Abstract

**Background/Objectives**: Peristomal skin complications are common among individuals with a stoma and are associated with decreased quality of life, increased healthcare costs, social isolation, and various other challenges. However, these complications can often be prevented through appropriate care, patient education, counseling, and follow-up. This quasi-experimental study aimed to evaluate the effectiveness of education based on Orem’s Self-Care Deficit Nursing Theory (SCDNT) in preventing peristomal skin complications. **Methods**: The study included 45 patients with newly formed stomas, allocated to an experimental group (n = 24) and a control group (n = 21) using a nonrandomized approach. Both groups received routine postoperative education, while the experimental group additionally received structured education, counseling, and follow-up based on SCDNT. Data were collected using the Patient Characteristics Form, Self-Care Agency Scale (SCAS), Stoma Quality of Life Scale (SQOL), Patient Outcomes Evaluation Form, and Patient Opinions Questionnaire. **Results**: Among the participants, 73.3% had undergone stoma surgery due to cancer, and 53.3% had an ileostomy. Peristomal skin complications were observed in 54.2% of patients in the experimental group and 95.2% in the control group (*p* < 0.05). The most frequently reported complications were irritant dermatitis (71.4%) and hyperplasia (22.7%). The mean recovery time was shorter in the experimental group (21 ± 12.95 days) compared to the control group (44.65 ± 23.56 days) (*p* < 0.05). **Conclusions**: SCDNT-based education, counseling, and follow-up may be associated with lower rates and shorter durations of peristomal skin complications and earlier patient engagement in self-care. However, these findings should be interpreted with caution due to the nonrandomized design, small sample size, and differences in follow-up intensity. Further randomized controlled studies are recommended to confirm these findings.

## 1. Introduction

Stoma formation is a common surgical procedure that helps manage fecal or urinary diversion, often due to conditions like colorectal cancer, inflammatory diseases, or trauma. While stomas can be life-saving and play a crucial role in maintaining essential bodily functions, they often come with a host of complications that can significantly impact a patient’s quality of life. Some of the typical issues include excessive gas, unpleasant odors, leakage, irritation of the skin around the stoma, indigestion, loss of appetite, nausea, diarrhea, constipation, and pain. These physical challenges can lead to psychological distress and social difficulties, such as anxiety, lowered self-esteem, and feelings of isolation [1,2,3,4,5].

Even with advancements in surgical techniques, medical technology, and the design of ostomy products, these complications still occur at a notable rate, with peristomal skin irritation being the most commonly reported issue. The reported prevalence of stomal and peristomal complications varies considerably across studies, reflecting differences in patient populations, follow-up duration, and care quality [1,4,5]. In Türkiye, available evidence remains limited but suggests that complication rates are substantial. However, Karadağ [6] retrospectively analyzed 128 patients with stomas who were regularly monitored in a stomatherapy unit and found a complication rate of 32.8%, with peristomal skin irritation making up 17.9% of those cases. In other national studies [2,7,8,9], the rates of peristomal complications have been reported to range from 5.8% to 48.7%. This reflects differences in care conditions and patient education levels. Importantly, access to specialized stomatherapy services is not uniformly available across the country, and the limited number of stomatherapy units may restrict patients’ access to structured education and follow-up care. In many small towns and cities there are no stomatherapy units and stomatherapy nurses. There are currently 35 stomatherapy units across Türkiye [10]. Although basic stoma care products (such as stoma bags, adapters, paste, powder and stoma caps) are widely available in medical supply shops in Türkiye, patients may face significant additional costs due to the difference between the amount reimbursed by the Social Security Institution and the market price. This situation may contribute to an increase in the incidence of complications. In centres where stomatherapy nurses provide care to patients, the standard care procedure involves informing the patient about the procedure prior to surgery and providing information on the stoma care products they will use post-surgery. Following the surgery, the patient and their relatives are given training on stoma care and the use of stoma care products. Information leaflets on living with a stoma are also provided.

It is important to note that nearly all patients with a stoma will likely face complications at some point. Numerous studies [5,9,11,12,13] have consistently indicated that peristomal skin complications are the most prevalent. Previous research has highlighted the importance of patient education in improving stoma-related outcomes. Educational interventions have been associated with reduced complication rates, shorter hospital stays, improved quality of life, and better adherence to care practices [12,14,15]. However, existing studies show variability in effectiveness, and many interventions lack a strong theoretical foundation, limiting their consistency and reproducibility. In particular, there is a lack of structured, theory-based educational models specifically targeting the prevention and management of peristomal skin complications.

Nurses play a vital role in the prevention and management of stoma-related complications. As members of a professional discipline dedicated to promoting health and providing holistic care, it is essential that nurses base their interventions on theoretical frameworks. Nursing theories offer structured guidance for clinical practice, enhance decision-making, and ensure consistency in care delivery. One such model is the Self-Care Deficit Nursing Theory (SCDNT), developed by Dorothea Orem. Orem posits that individuals can develop the necessary intellectual and practical skills to manage their own care if properly motivated and educated. SCDNT provides a comprehensive framework for supporting individuals in meeting their self-care needs. The theory emphasizes that individuals can develop the cognitive and psychomotor skills necessary for effective self-care when provided with appropriate guidance, support, and education. In the context of stoma care, SCDNT offers a structured approach to enhancing patients’ ability to perform essential tasks such as proper skin care, correct use of ostomy appliances, early recognition of complications, and timely management of emerging problems [16].

Importantly, these mechanisms are directly relevant to the prevention of peristomal skin complications. Inadequate appliance fitting, poor hygiene practices, delayed recognition of skin irritation, and improper product use are well-documented contributors to skin problems. A theory-based educational intervention grounded in SCDNT may improve patients’ self-efficacy, promote accurate and consistent care practices, and facilitate early intervention, thereby reducing both the incidence and severity of complications.

Despite this theoretical relevance, there is limited empirical evidence examining the effectiveness of SCDNT-based education specifically on peristomal skin outcomes. Addressing this gap is particularly important in healthcare settings where access to specialized stomatherapy support is limited. Therefore, this study aims to evaluate the effectiveness of an SCDNT-based educational intervention on clinically relevant outcomes in patients with newly created stomas.

### Aims and Research Questions

This quasi-experimental study aimed to evaluate the effectiveness of education based on the SCDNT in preventing and managing peristomal skin complications among patients with newly created stomas.

The research questions were as follows:

1. Is SCDNT-based education effective in reducing the incidence of peristomal skin complications?
2. Is SCDNT-based education effective in reducing the healing time of peristomal skin complications?
3. Is SCDNT-based education effective in increasing patients’ participation in stoma care?

Hypotheses:

**H1:**  *Patients receiving SCDNT-based education will experience a lower incidence of peristomal skin complications compared to those receiving standard care.*

**H2:**  *Patients in the intervention group who develop peristomal skin complications will demonstrate shorter healing times compared to the control group.*

**H3:**  *Patients receiving SCDNT-based education will demonstrate higher levels of participation in stoma care compared to those receiving standard care.*

The selected outcomes reflect key dimensions of stoma care, including complication prevention, clinical recovery, and patient engagement. These dimensions are consistent with the core assumptions of SCDNT, which emphasizes the development of patients’ self-care agency through structured education and support.

## 2. Materials and Methods

### 2.1. Research Design

This quasi-experimental, non-randomized study was conducted in the General Surgery Clinic and Stomatherapy Unit of a University Health Research and Application Center, where approximately 100 stoma surgeries are performed annually. The study duration was 8 months.

### 2.2. Sample and Power Analysis

The study population comprised patients with a newly created stoma treated at the study center during the data collection period. A total of 45 patients (intervention: n = 24; control: n = 21) who met the inclusion criteria were included.

Inclusion criteria were: age ≥ 18 years, newly created stoma, ability to read and write in Turkish, access to the internet, absence of physical or psychiatric conditions preventing self-care, and willingness to participate.

For the purpose of power estimation, the primary endpoint was defined as the incidence of peristomal skin complications during the follow-up period, as this represents the most clinically relevant outcome of the study.

An a priori power analysis was conducted using PASS 2023. Assuming a moderate effect size (Cohen’s d = 0.5), α = 0.05, and 80% power, a minimum sample size of 42 participants (21 per group) was estimated. The assumed effect size was based on a pragmatic expectation of moderate effects observed in prior educational and follow-up interventions targeting complication-related and self-care outcomes; however, it was not derived from a single definitive prior study and should be interpreted as an approximation.

Given that approximately 100 stoma creation procedures are performed annually at the study center, the sample size was also informed by feasibility constraints within a single-center design.

During the study period, 69 patients were assessed for eligibility; 24 were excluded due to refusal (n = 5), dropout (n = 4), lack of internet access (n = 5), illiteracy (n = 4), or clinical deterioration (n = 6).

Given the relatively small sample size and the assumptions underlying the effect size, the study may be underpowered for secondary outcomes, subgroup analyses, and repeated-measures comparisons. Therefore, the findings should be interpreted with caution, particularly for outcomes beyond the primary endpoint. Furthermore, the inclusion criteria—particularly the requirement for literacy and internet access—may have introduced selection bias and limit the generalizability of the findings to more vulnerable patient populations.

### 2.3. Group Assignment

Participants were allocated to intervention and control groups using an alternating two-week block design. Patient-level randomization was not feasible due to the risk of contamination, as the intervention involved structured education and follow-up delivered by the same clinical team. In addition, shared staffing and workflow constraints limited the implementation of concurrent parallel protocols.

Although temporal separation reduced contamination risk, it may have introduced temporal confounding. To minimize this, both groups were recruited within the same study period under similar clinical conditions, and baseline characteristics were assessed for comparability. Nevertheless, residual confounding related to time-dependent factors (e.g., case-mix or staffing variations) cannot be excluded.

### 2.4. Ethical Considerations

Ethical approval was obtained from the hospital (13.02.2017–E-22427) and Ethics Committee (21.04.2017–E.59760). Written informed consent was obtained from all participants after providing detailed information about the study procedures. Participants were informed about the purpose, scope, and handling of photographic data, and specific consent was obtained for the capture and use of these images. All images were transmitted via secure, password-protected channels and were stored on encrypted devices accessible only to the research team.

To ensure confidentiality, all photographs were de-identified prior to analysis, and no personally identifiable information was stored alongside the images. Data were handled in accordance with institutional data protection policies, and all digital materials were securely stored and used solely for research purposes.

### 2.5. Data Collection Tools

The following instruments were used: Patient Characteristics Form (PCF), Self-Care Agency Scale (SCAS) [17], Stoma Quality of Life Scale (SQOL) [18], Peristomal Skin Complications Management Training Booklet (PSCMTB), Patient Outcomes Evaluation Form (POEF), and Patient Opinions Questionnaire (POQ). Patients also submitted peristomal photographs taken with mobile devices throughout follow-up. The content validity of researcher-developed tools (PCF, PSCMTB, POEF, POQ) was assessed by 11 experts, yielding a content validity index of 1.0.

#### 2.5.1. Patient Characteristics Form (PCF)

The PCF comprises 33 items across two sections: sociodemographic data (13 items, e.g., age, sex, education, comorbidities) and stoma-related information (20 items, e.g., diagnosis, surgery type, stoma site marking, early complications).

#### 2.5.2. Self-Care Agency Scale (SCAS)

Originally developed by Kearney and Fleischer in 1979 and adapted by Nahcivan (1994) [17] for Turkish populations, the SCAS contains 35 items measuring self-care ability. Responses range from 0 (“does not define me at all”) to 4 (“defines me a lot”), with reverse scoring applied to eight (3, 6, 9, 13, 19, 22, 26 and 31) negatively worded items. The scale’s Cronbach’s alpha reliability coefficient is 0.92 [17].

#### 2.5.3. Stoma Quality of Life Scale (SQOL)

Developed by Baxter et al. [19] and validated in Turkey by Karadağ et al. [18], the SQOL has 21 items divided into four subdimensions: work/social life, sexuality/body image, stoma function, plus two standalone items assessing overall life satisfaction and economic status. Items use a 5-point Likert scale, and subdimensions are scored from 0 to 100, with higher scores indicating better quality of life.

#### 2.5.4. Peristomal Skin Complications Management Training Booklet (PSCMTB)

**Figure 1:**
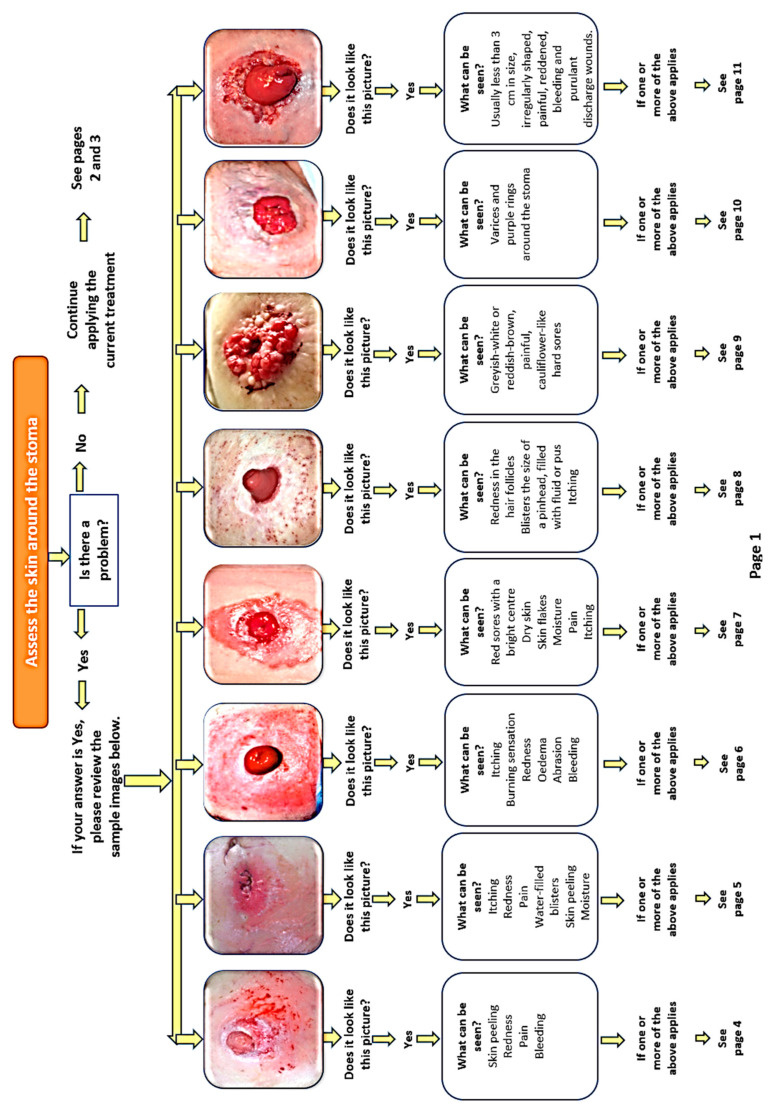
The first stage of determining the type of complication.

**Figure 2:**
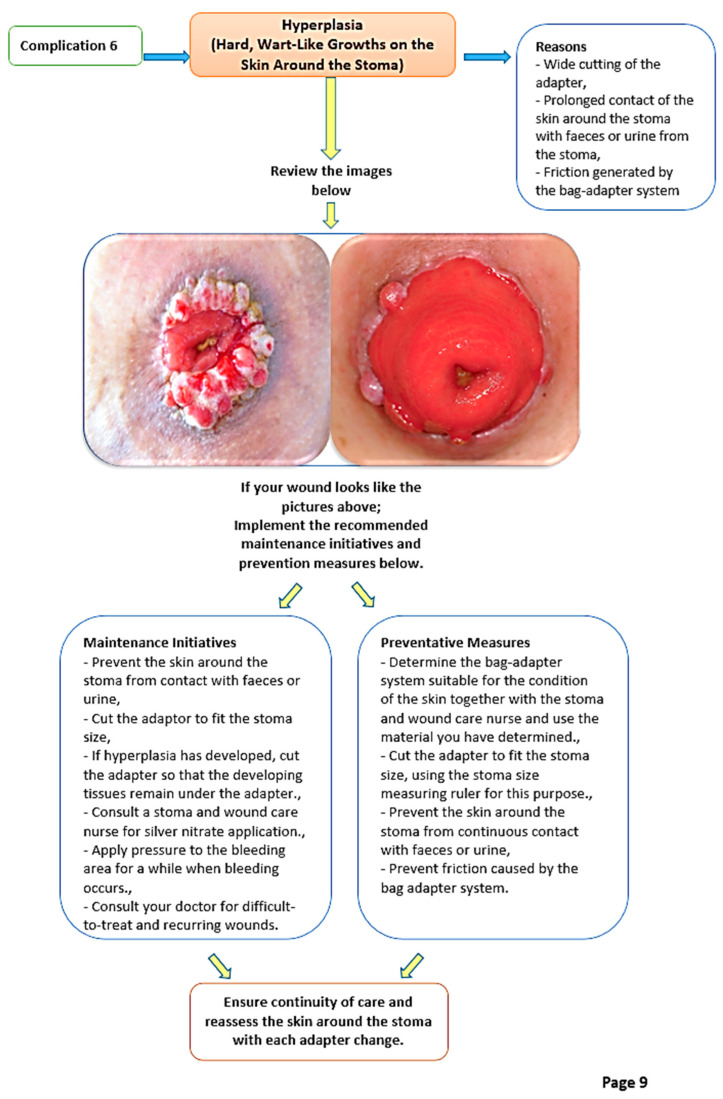
Verification of the type of complication with different sample pictures and causes.

**Figure 3:**
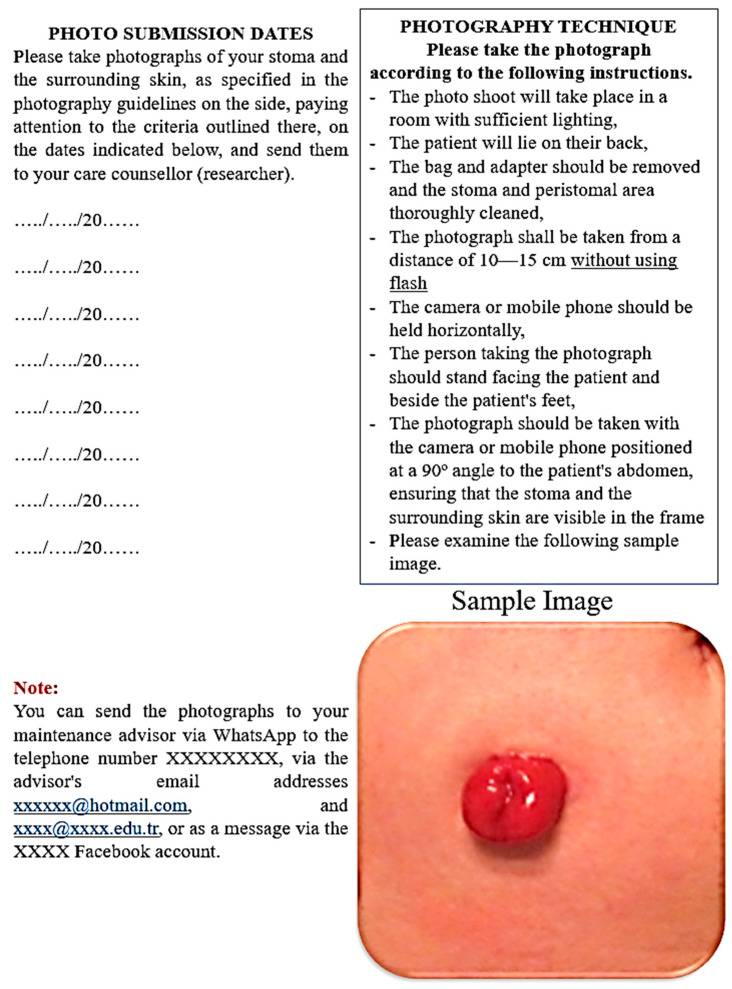
Instructions for taking and sending photos.

The PSCMTB, developed based on relevant literatüre [4,9,11,20], addresses eight common peristomal skin complications with sample images, symptom descriptions, care interventions, and prevention guidelines. The booklet is divided into three parts: assessment algorithms (Figure 1 and Figure 2) for peristomal skin, routine stoma care instructions, and management strategies for complications. It also includes guidance on photography techniques, photo submission schedules, and researcher contact details (Figure 3).

#### 2.5.5. Patient Outcomes Evaluation Form (POEF)

This nine-item form records data collected during the three-month follow-up, including caregiver involvement, patient participation in stoma care, patient’s goals of participation in care, presence of complications, counseling provided, care recommendations, hospitalizations, and recovery status.

#### 2.5.6. Patient Opinions Questionnaire (POQ)

Designed to evaluate patient feedback on the PSCMTB, the POQ uses an 11-item, 5-point Likert scale (1 = strongly disagree to 5 = strongly agree) assessing the booklet’s clarity, content adequacy, visual appropriateness, usability, and its role in promoting patient engagement. It also contains an open-ended question for additional comments.

### 2.6. Implementation

Preoperative stoma education and site marking were provided by a stomatherapy nurse using a standardized booklet. After surgery, all participants were informed about the study and initial assessments (PCF, SCAS) were completed. Patients received care according to group assignment: SCDNT-based care for the experimental group, routine hospital care for the control group. All participants were trained in stoma self-care and discharged with educational materials.

All participants received routine discharge education on stoma self-care and standard written educational materials in accordance with usual clinical practice.

The control group received standard care only, which included routine education at discharge and scheduled follow-up through peristomal photograph submission without structured feedback beyond usual clinical responses.

The intervention group received an enhanced, nurse-led self-care program grounded in Orem’s SCDNT. In addition to standard care, this program included (i) structured and reinforced education sessions, (ii) use of a supportive educational booklet, and (iii) regular tele-follow-up with individualized feedback based on submitted peristomal photographs.

Thus, the intervention represented an intensified, theory-informed and follow-up-supported approach rather than a standalone educational component.

The frequency and mode of contact differed between groups: while both groups submitted photographs at similar intervals, only the intervention group received systematic feedback and guidance. The potential influence of monitoring itself on patient behavior was considered in the interpretation of findings.

Due to the multi-component nature of the intervention, the effects observed in this study should be interpreted as reflecting the combined impact of education, reinforcement, and tele-support, rather than any single element in isolation.

According to the literature [1,2,5,8], early complications occur within the first month, and late complications typically appear 6–10 weeks after surgery. Therefore, a 3-month (90-day) follow-up period was selected to observe both early and late peristomal skin complications in patients with permanent and temporary stomas.

#### 2.6.1. Experimental Group Protocol

In addition to standard care, experimental group patients received PSCMTB-based education aligned with SCDNT framework. SCAS and SQOL were administered pre- and post-intervention. Patients submitted photographs of the peristomal area weekly during the first month, biweekly in the second month, and monthly in the third month. Remote support and follow-up were provided via the booklet and telephone. Interventions focused on correcting incorrect care, promoting self-care behavior, setting progressive goals, and providing support through the stomatherapy unit when needed. After 90 days, SCAS, SQOL, and POQ were re-administered.

#### 2.6.2. Control Group Protocol

Control group patients received standard care and education and completed the same pre- and post-discharge assessments (PCF, SCAS, SQOL). They followed the same photo submission schedule and were evaluated similarly via POEF. Care support was limited to routine follow-up and consultation when needed.

#### 2.6.3. Pilot Study

A preliminary implementation was conducted with 10 patients (5 per group). As no issues were encountered, these participants were included in the main study.

#### 2.6.4. Data Collection Standardization

A structured “Interview Steps Checklist for the Researcher” was used throughout the study to ensure consistency across both groups. The checklist was designed to standardize follow-up procedures, minimize omissions, and ensure uniform data collection. It was reviewed for content validity by a researcher with expertise in stoma care.

For outcome adjudication, patient-submitted stoma photographs were evaluated using a predefined checklist based on observable clinical indicators (e.g., peristomal skin condition, presence of irritation, leakage signs). All photographs were assessed by a single trained researcher who was familiar with the checklist criteria. Due to the nature of the intervention and follow-up process, the assessor was not blinded to group allocation.

An independent second assessor was not used; therefore, inter-rater reliability could not be evaluated. To mitigate potential assessment bias, all evaluations were conducted using explicit checklist criteria, and ambiguous cases were re-reviewed by the same assessor after a time interval to enhance intra-rater consistency.

Healing time was operationally defined as the number of days from discharge to the first follow-up point at which the peristomal skin was assessed as intact, with no visible irritation, redness, or breakdown according to the checklist criteria.

Participation in stoma care was defined based on patient self-reported involvement during follow-up interviews and categorized into three levels: (1) full self-care (independent performance of all stoma-related tasks), (2) partial participation (performing some tasks with assistance), and (3) no participation (care fully performed by a caregiver). These categories were assigned according to predefined criteria to ensure reproducibility.

### 2.7. Statistical Analysis

All data were analyzed using SPSS for Windows, version 22.0. Descriptive statistics (mean, standard deviation, frequency, and percentage) were used to summarize patient characteristics and outcome measures. A significance level of *p* < 0.05 was considered statistically significant.

To examine associations between categorical variables and complication development, Fisher’s exact test, likelihood ratio test, or Pearson’s chi-square test were applied as appropriate. Changes in patient participation in care over time were assessed using Fisher’s exact test, Pearson’s chi-square test, and Cochran’s Q test.

The Mann–Whitney U test was used to compare SCAS and SQOL scores between groups, as data were non-normally distributed and group sizes were less than 30 (experimental = 24, control = 21). Within-group comparisons were conducted using the Wilcoxon signed-rank test due to non-normal distribution and repeated measurements.

No formal adjustment for multiple comparisons was applied, and secondary analyses should be interpreted as exploratory.

## 3. Results

Most stomas were created due to colorectal cancer, and nearly all patients underwent planned surgery. Stoma site marking was performed preoperatively in 75% of the experimental group and 71.4% of the control group. More than half had temporary stomas, and ileostomy was the most common type (Table 1 and Table 2).

Within the first 24 h postoperatively, 15.6% of patients experienced at least one complication.

At the patient level, by the end of the three-month follow-up, 54.2% of patients in the experimental group and 95.2% in the control group (Table 3) had experienced at least one complication (*p* < 0.05). Statistically significant differences between groups emerged at the 2nd, 3rd, and 4th follow-ups, consistently showing lower proportions of affected patients in the experimental group (*p* < 0.05). No new patients developed complications in the experimental group after the 4th follow-up.

At the episode level, a total of multiple complication events were recorded across follow-up periods, reflecting repeated occurrences in some patients. Some patients experienced more than one complication over time, and episode counts exceeded the number of unique patients.

The most frequently observed complication was irritant contact dermatitis, accounting for 71.4% of all recorded complication episodes, followed by hyperplasia (22.7%) (Table 4).

Regarding associated factors, stoma type, output level, and stoma positioning were significantly associated with complication occurrence at specific follow-ups. Higher complication rates were observed in patients with ileostomy at the 2nd follow-up, those with excessive output at the 4th follow-up, and those with stomas at or below skin level at the 1st follow-up (*p* < 0.05).

**Figure 4:**
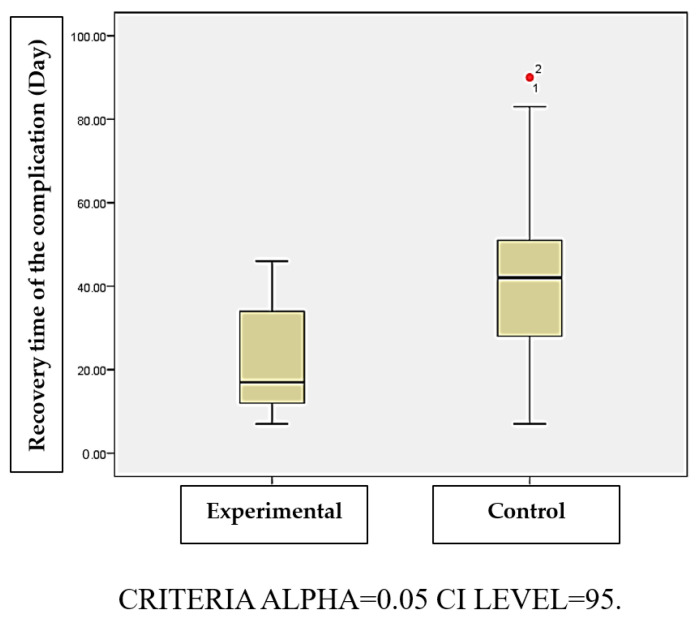
Boxplot of recovery times associated with peristomal skin complications.

The average complication recovery time was shorter in the experimental group (21 ± 12.95 days) than in the control group (44.65 ± 23.56 days) (*p* < 0.05) (Table 5, Figure 4).

At the 1st follow-up, more patients in the experimental group participated in stoma care (*p* < 0.05). Participation increased over time in both groups (*p* < 0.05), with earlier improvement in the experimental group. By the 4th follow-up, 33.3% of the experimental group and 19.0% of the control group could independently manage care, though this difference was not significant (*p* > 0.05).

The control group received more counseling overall, mostly by phone, and experienced a sharp increase in complications at the 2nd follow-up—likely due to transitioning to home care. The most frequent advice given was to properly size the appliance and protect exposed skin. The control group also had more hospital admissions due to complications. These findings partially support the hypothesis that SCDNT-based training increases self-care participation.

No significant differences were found in pretest–posttest SCAS or SQOL scores between the groups (*p* > 0.05). Improvements in SQOL subdimensions were also not significant, except for work/social life (all patients) and sexuality (control group) (*p* < 0.05). These findings do not support the idea that SCDNT-based training improves self-care agency or quality of life scores.

Most patients found the booklet (PSCMTB) beneficial for self-care, informative, easy to understand, and useful in managing skin issues. They reported that it helped reduce hospital admissions and was easy to use. Common responses included: “It was very useful” (70.8%), “I think it is necessary” (79.2%), and “It was nice to have someone to consult.”

## 4. Discussion

The findings of this study suggest that a structured, theory-informed, and follow-up–intensive educational approach was associated with lower rates and shorter duration of peristomal skin complications, as well as earlier engagement in self-care activities. However, these findings should be interpreted cautiously, as the intervention represents a multi-component care package—including education, tele-monitoring, reinforcement, and increased contact—rather than an isolated test of SCDNT. These outcomes align with previous literature, which highlights the importance of structured patient education in reducing postoperative complications and improving care outcomes [2,8,15].

Importantly, the observed benefits cannot be attributed solely to SCDNT. Increased surveillance, more frequent interaction, and timely feedback likely played a significant role. Therefore, SCDNT should be considered a guiding framework within a broader intervention rather than the primary causal mechanism.

In this study, 15.6% of patients developed early complications within the first 24 h postoperatively, with irritant contact dermatitis being the most common diagnosis—consistent with earlier reports [1,7,8,14]. During the 3-month follow-up, the cumulative complication rate was lower in the experimental group than in the control group. No new complications were observed in the experimental group at the final follow-up, this finding suggests that the intervention, which was delivered within a structured, theory-informed framework, and embedded in a multi-component follow-up program, may be associated with this outcome.

The most prevalent complications—irritant contact dermatitis (71.4%) and hyperplasia (22.6%)—mirror those reported in prior studies [1,4,9]. The observed increase in complications during the immediate post-discharge period (second follow-up) likely reflects the transition from supervised hospital care to independent home care. However, this trend was less pronounced in the experimental group, likely due to continued structured follow-up and guidance based on the PSCMTB. This supports previous findings that highlight the importance of continuity of care and targeted education in the early postoperative period [4,5,14].

The mean recovery time for complications was shorter in the experimental group (21 ± 12.95 days) compared to the control group (44.65 ± 23.56 days). Although literature lacks robust data on recovery duration, the shorter recovery time observed in this study may reflect earlier identification, closer follow-up, and timely management of complications within the multi-component intervention rather than a direct effect of theory-based education alone.

Another finding was the increased and earlier participation in stoma care observed in the experimental group. While patients in the experimental group engaged in self-care earlier, the lack of a sustained between-group difference at later follow-ups suggests that the intervention may have influenced the timing of engagement rather than long-term independence. Previous studies have shown that early patient involvement in self-care leads to improved confidence, reduced dependency, and better overall adjustment [5,12,21]. The educational approach used in this study emphasized collaborative goal setting, motivational interviewing, and reinforcement—all of which have been identified as effective strategies in promoting self-care among patients with chronic health conditions [15,21].

Regarding self-care engagement, patients in the experimental group began participating in stoma care earlier and more consistently. While both groups showed increased participation over time, the experimental group demonstrated a statistically significant earlier transition to independent care. This aligns with the conceptual framework of SCDNT, which emphasizes guided support during periods of self-care deficit and gradual restoration of autonomy [16]. Previous studies have shown that enhanced education improves care participation and adaptation [15,21]. In addition, Ko et al. reported that multimedia training given to patients with stoma significantly increased patients’ self-care skills and QoL scores. Encouraging and supporting patients to participate in care is very effective in increasing adaptation [21].

Although improvements in SCAS and overall SQOL scores were observed, these changes were not statistically significant. However, subdimension analysis of the SQOL revealed improvements in the work/social life domain across both groups and in sexuality/body image for the control group. The absence of significant improvement in the experimental group may reflect heightened awareness of potential complications resulting from increased education, as well as ongoing psychosocial distress related to cancer diagnoses and adjuvant therapies. Similar to prior findings [1,12,20], patients experienced multifaceted challenges—physical, psychological, and social—that may have negatively affected QoL outcomes despite reduced complication rates. These observations support the notion that a longer follow-up period may be necessary to capture the full benefits of educational interventions on self-care capacity and QoL. It is also possible that ongoing cancer treatments and psychological distress related to stoma adjustment diminished the impact of the educational intervention on broader QoL indicators.

Nevertheless, patient feedback on the PSCMT booklet was overwhelmingly positive. The majority found it useful, easy to understand, and supportive in daily self-care, suggesting that theory-based, structured educational materials can enhance patient experience and promote engagement. The booklet’s emphasis on self-care goals, complication recognition, and problem-solving may be associated with hospital admissions and complication severity. Educational materials developed using a theoretical framework and tailored to patient needs have been found to enhance knowledge retention and improve care behaviors [15].

In summary, these findings suggest that a structured, theory-informed, and follow-up–intensive approach may be associated with improved clinical outcomes and earlier engagement in care; however, these effects should be interpreted as reflecting the combined influence of multiple intervention components rather than the independent effect of SCDNT. While long-term psychosocial adaptation remains a challenge, especially in oncology populations, early structured support appears essential to promote effective self-care and reduce complications.

### Research Limitations

This study has several limitations. First, the quasi-experimental design with time-based group allocation introduces risks of selection bias and temporal confounding. Second, the small sample size limits statistical power, particularly for secondary outcomes and subgroup analyses. Third, lack of blinding and reliance on patient-submitted photographs may have introduced detection and classification bias. Fourth, the intervention consisted of multiple components—including structured education, tele-follow-up, and reinforcement—making it difficult to isolate the specific contribution of SCDNT. Fifth, researcher-developed tools lacked comprehensive psychometric validation within this sample. Sixth, the absence of multivariable analysis limits the ability to control for potential confounders such as stoma type or output characteristics.

In addition, pilot participants were retained in the final sample, which may have introduced bias due to prior exposure to study procedures and intervention elements.

Furthermore, no formal adjustment for multiple comparisons was applied despite repeated testing across follow-up points and outcomes; therefore, the risk of type I error is increased, and secondary findings should be interpreted as exploratory.

Finally, inclusion criteria requiring literacy and internet access may limit generalizability to more vulnerable patient populations. Overall, findings should be interpreted as preliminary and hypothesis-generating rather than definitive.

## 5. Conclusions

This study suggests that a structured, theory-informed educational and follow-up approach may be associated with reduced incidence and shorter duration of peristomal skin complications, as well as earlier patient engagement in care. However, given the nonrandomized design, small sample size, and the multi-component nature of the intervention, these findings should be interpreted with caution.

The results do not allow attribution of effects specifically to SCDNT but rather indicate the potential value of combining structured education with ongoing support and monitoring. Further well-designed randomized controlled trials with larger samples, standardized interventions, and longer follow-up are needed to confirm these findings and clarify underlying mechanisms.

## Figures and Tables

**Table 1:** Sociodemographic Characteristics of The Patients (n = 45).

	Experiment (n = 24) n (%)	Control (n = 21) n (%)	Statistical Analysis
Age			
X ± SD (min–max)	57.04 ± 11.07 (34–75)	53.66 ± 13.11 (18–72)	U = 213.000 *p* = 0.374 ***
Gender			
Female	12 (50.0)	9 (42.9)	X^2^ = 0.632
Male	12 (50.0)	12 (57.1)	*p* = 0.767 ****
Clinic			
General Surgery	18(75.0)	19(90.5)	X^2^ = 0.176
Urology	6(25.0)	2(9.5)	*p* = 0.252 ****
Chronic Disease			
Yes	10(41.7)	9(42.9)	X^2^ = 0.936
No	14(58.3)	12(57.1)	*p* = 1.000 ****
Receiving Additional Treatment (CT, RT, CAM)			
Yes	10 (41.7)	14 (66.7)	X^2^ = 0.094
No	14 (58.3)	7 (33.3)	*p* = 0.136 ****
Marital Status			
Married	22 (91.7)	20 (95.2)	X^2^ = 0.632
Single	2 (8.3)	1 (4.8)	*p* = 1.000 ****
Education Status			
Primary school or below **	14 (58.3)	12 (57.1)	X^2^ = 0.926
Middle School	3 (12.5)	2 (9.5)	
High school and above	7 (29.2)	7 (33.3)	
Job			
Retired	12 (50.0)	8 (38.1)	X^2^ = 0.891
Housewife	10 (41.7)	5 (23.8)	*p* = 0.555 ****
Officer	2 (8.3)	3 (14.3)	
Tradesmen	0 (0.0)	1 (4.8)	
Student	0 (0.0)	1 (4.8)	
Not working or Self Employed	0 (0.0)	3 (14.3)	
Lifestyle			
Active	9 (37.5)	10 (47.6)	X^2^ = 0.493
Sedanter	15 (62.5)	11 (52.4)	
Social Life			
Going out	15 (62.5)	9 (37.5)	X^2^ = 0.967
Spending time at home	13 (61.9)	8 (38.1)	*p* = 1.000 ****
Place of Residence			
City	19 (79.2)	15 (71.4)	X^2^ = 0.547
District/Town/Village	5 (20.8)	6 (28.6)	*p* = 0.730 ****

CT: Chemotherapy, RT: Radiotherapy, CAM: Complementary and Alternative Medicine. ** Primary school graduate or literate.*** Mann–Whitney U Test was used. **** Fisher’s Exact Test was used.

**Table 2:** Patients’ Characteristics Related to The Stoma (n = 45).

	Experiment (n = 24) n (%)	Control (n = 21) n (%)	Statistical Analysis
Medical diagnosis			
Cancer	17 (70.8)	16 (76.2)	X^2^ = 0.891
Inflammatory Bowel Disease	1 (4.2)	1 (4.8)	
Other *	6 (25.0)	4 (19.0)	
Type of Surgery			
Elective	19 (79.2)	20 (95.2)	X^2^ = 0.114
Urgent	5 (20.8)	1 (4.8)	*p* = 0.193 **
Stoma Area Marking			
Yes	18 (75.0)	15 (71.4)	X^2^ = 0.787
No	6 (25.0)	6 (28.6)	*p* = 0.1000 **
Duration of the stoma			
Permanent	8 (33.3)	5 (23.8)	X^2^ = 0.482
Temporary	16 (66.7)	16 (76.2)	*p* = 0.528 **
Type of stoma			
Colostomy	8 (33.3)	5 (23.8)	X^2^ = 0.205
Ileostomy	10 (41.7)	14 (66.7)	
Urostomy	6 (25.0)	2 (9.5)	
Shape of the stoma			
Round	9 (37.5)	11 (52.4)	X^2^ = 0.316
Oval or shapeless	14 (58.3)	8 (38.1)	*p* = 0.377 **
Soma Height			
Bud	19 (79.2)	13 (61.9)	X^2^ = 0.202
Same level with the skin or retracted	5 (20.8)	8 (38.1)	*p* = 0.323 **
Output Type			
Liquid	22 (91.7)	20 (95.2)	X^2^ = 0.632
Solid	2 (8.3)	1 (4.8)	*p* = 1.000 **
Output Quantity			
Too much (>2000 mL/24 h)	0 (0.0)	3 (14.3)	X^2^ = 0.109
Normal (400–800 mL/24 h)	23 (95.8)	18 (85.7)	
Very little (<400 mL/24 h)	1 (4.2)	0 (0.0)	
Presence of folds in the peristomal area			
Yes	8 (33.3)	8 (38.1)	X^2^ = 0.739
No	16 (66.7)	13 (61.9)	*p* = 0.765 **
Bag-Adapter System Used			
One-piece-flat adapter	2 (8.3)	0 (0.0)	X^2^ = 0.193
Two-piece-flat adapter	19 (79.2)	15 (71.4)	
Two-piece convex adapter	3 (12.5)	6 (28.6)	

* Intestinal obstruction, Intestinal perforation, Mucocutaneous Fistula. ** Fisher’s Exact Test was used.

**Table 3:** Patient-Level Complication Rates.

Outcome	Experimental (n, %)	Control (n, %)	*p*-Value
≥1 complication (3 months)	13 (54.2%)	20 (95.2%)	<0.05

**Table 4:** Peristomal Skin Compications Across Follow-Up: Episode-Level Distribution.

Complication Type	1st Follow-Up (15th Day)			2nd Follow-Up (1st Month)			3rd Follow-Up (2nd Month)			4th Follow-Up (3rd Month)			Total Episodes		
	Exp	Ctrl	Total	Exp	Ctrl	Total	Exp	Ctrl	Total	Exp	Ctrl	Total	Exp n (%)	Ctrl n (%)	Total n (%)
Allergic Contact Dermatitis	1	0	1	0	1	1	0	2	2	0	0	0	1 (3.23)	3 (3.40)	4 (3.36)
Irritant Contact Dermatitis	7	16	23	15	25	40	5	14	19	0	3	3	27 (87.09)	58 (65.90)	85 (71.42)
Peristomal Trauma	0	0	0	0	0	0	0	1	1	0	0	0	0 (0.00)	1 (1.14)	1 (0.84)
Hyperplasia	2	2	4	0	5	5	1	15	16	0	4	4	3 (9.68)	26 (27.28)	29 (22.7)
Uric Acid Crystals	0	0	0	0	1	1	0	0	0	0	1	1	0 (0.00)	2 (2.28)	2 (1.68)
Total Episodes	10	18	28	15	32	47	6	32	38	0	8	8	31 (100.0)	90 (100.0)	121 (100.0)

Exp: Experimental, Ctrl: Control. Values represent the number of complication episodes observed at each follow-up. Because some patients experienced more than one complication over time, the total number of episodes exceeds the number of unique patients. Percentages in the ‘Total Episodes’ column are calculated based on the total number of complication episodes within each group.

**Table 5:** Healing Time of Peristomal Skin Complications (days).

Group	Average	Std. Deviation	Minimum	Maximum	*p*
Experiment	21.00	12.95505	7.00	46.00	0.002 *
Control	44.65	23.56240	7.00	90.00	

* Mann–Whitney U Test was used.

## Data Availability

The data presented in this study are available on request from the corresponding author due to (The institution where the study was conducted does not permit the sharing of records with other individuals, institutions, or organizations).

## Public Involvement Statement

Patients were informed about the study, and their consent was obtained. It was explained that participation was voluntary and that they could withdraw from the study at any time without providing a reason. They were assured that the collected information would remain confidential and would not be used for any other purpose. Following this information, written informed consent was obtained from the patients who agreed to participate. All stages of the study were completed in accordance with the principles of the Declaration of Helsinki.

## Guidelines and Standards Statement

No reporting guideline were used when drafting the report.

## Abbreviations

The following abbreviations are used in this manuscript:

SCDNT	Self-Care Deficit Nursing Theory
SCAS	Self-Care Agency Scale
SQOL	Stoma Quality of Life Scale
PCF	Patient Characteristics Form
PSCMTB	Peristomal Skin Complications Management Training Booklet
POEF	Patient Outcomes Evaluation Form
POQ	Patient Opinions Questionnaire
